# The End of the Cold Loneliness: 3D Comparison between *Doto antarctica* and a New Sympatric Species of *Doto* (Heterobranchia: Nudibranchia)

**DOI:** 10.1371/journal.pone.0157941

**Published:** 2016-07-13

**Authors:** Juan Moles, Heike Wägele, Manuel Ballesteros, Álvaro Pujals, Gabriele Uhl, Conxita Avila

**Affiliations:** 1 Department of Evolutionary Biology, Ecology, and Environmental Sciences and Biodiversity Research Institute (IrBIO), University of Barcelona, Av. Diagonal 645, 08028 Barcelona, Catalonia, Spain; 2 Zoological Research Museum Alexander Koenig, Adenauerallee 160, 53113 Bonn, Germany; 3 General and Systematic Zoology, Zoological Institute and Museum, University of Greifswald, Anklamer Str. 20, 17489 Greifswald, Germany; Nanjing University, CHINA

## Abstract

Although several studies are devoted to determining the diversity of Antarctic heterobranch sea slugs, new species are still being discovered. Among nudibranchs, *Doto antarctica* Eliot, 1907 is the single species of this genus described from Antarctica hitherto, the type locality being the Ross Sea. *Doto antarctica* was described mainly using external features. During our Antarctic research on marine benthic invertebrates, we found *D*. *antarctica* in the Weddell Sea and Bouvet Island, suggesting a circumpolar distribution. Species affiliation is herein supported by molecular analyses using cytochrome *c* oxidase subunit I, 16S rRNA, and histone H3 markers. We redescribe *D*. *antarctica* using histology, micro-computed tomography (micro-CT), and 3D-reconstruction of the internal organs. Moreover, we describe a new, sympatric species, namely *D*. *carinova* Moles, Avila & Wägele n. sp., and provide an anatomical comparison between the two Antarctic *Doto* species. Egg masses in both species are also described here for the first time. We demonstrate that micro-CT is a useful tool for non-destructive anatomical description of valuable specimens. Furthermore, our high resolution micro-CT data reveal that the central nervous system of both *Doto* species possesses numerous accessory giant cells, suggested to be neurons herein. In addition, the phylogenetic tree of all *Doto* species sequenced to date suggests a scenario for the evolution of the reproductive system in this genus: bursa copulatrix seems to have been reduced and the acquisition of a distal connection of the oviduct to the nidamental glands is a synapomorphy of the Antarctic *Doto* species. Overall, the combination of thorough morphological and anatomical description and molecular analyses provides a comprehensive means to characterize and delineate species, thus suggesting evolutionary scenarios.

## Introduction

Heterobranch sea slugs are worldwide-distributed molluscs with new species being discovered regularly from tropical and temperate areas, while Polar Regions are less explored [1]. Interestingly, basal members of some major Nudipleura and Pleurobranchomorpha linages have an Antarctic origin [2–4]. Among nudibranchs, the family Dotidae Gray, 1853 is presently considered to be a monophyletic taxon within Cladobranchia [5]. However, the relationships between Dotidae and the other cladobranch families remain undefined [6], although RNAseq analyses of Goodheart *et al*. [7] indicate a closer relationship to Dendronotida. In fact, Dotidae was traditionally placed into Dendronotida (formerly Dendronotoidea) [8] based on the presence of rhinophoral sheaths, into which the rhinophores can be retracted [9]. A cuticle lining of the stomach and tentacular expansions of the oral veil were lately advocated as additional autapomorphies of Dendronotida [10]. Since these traits are not present in Dotidae, their systematic position within Dendronotida is questionable.

Dotidae comprises four genera: *Doto* Oken, 1815, *Caecinella* Bergh, 1870, *Miesea* Marcus, 1961, and the recently described *Kabeiro* Shipman & Gosliner, 2015. However, the taxonomic status of the monotypic *Caecinella* and *Miesea* is often considered doubtful [9,11]. *Doto* differs from *Miesea* in having rhinophoral sheaths [12], and from *Kabeiro* by the shape and arrangement of the cerata, the pericardium size, and the absence of a penial gland [13]. *Doto*, with 87 species recognised to date [14] shows a cosmopolitan distribution, and the species are usually defined only on the basis of external characters, *i*.*e*., colouration, number and shape of cerata, and shape of the rhinophoral sheath [15]. Lemche [16] took a wider approach including not only body colour pattern, but also food preference and shape of the egg mass. However, information on anatomical characters of the digestive, reproductive, circulatory, nervous, or excretory systems for most *Doto* species remains poorly known.

Only *Doto antarctica* Eliot, 1907 has been described from Antarctica to date, based on a single specimen from McMurdo Sound (Victoria Land) [17]. A brightly-yellow coloured species of *Doto* was recorded from the Davies Sea (eastern Antarctica) and roughly described by Thiele [18]. However, the material was insufficient to properly describe the species. Later, Odhner [19] added details of the external anatomy and radula of *D*. *antarctica* from Cape Adare (Victoria Land) but did not provide internal description of the digestive and reproductive systems. Since then, *D*. *antarctica* has been found in King George Island (South Shetland Islands) at 160 m [20] and in the Victoria Land (Ross Sea) at 80–500 m depth [21,22]. Additional undetermined species of *Doto* have been recorded in Bouvet Island [23] and the Ross Sea [22,24].

Here, we use histological and tomographic techniques to explore the organ systems and the egg masses of *D*. *antarctica*. In addition, we newly describe a single specimen of *Doto carinova* n. sp., collected in the Weddell Sea, by 3D reconstruction of micro-CT images. Thereby, we assessed the potential of micro-CT for non-invasive description of singleton type material [25,26]. Moreover, we sequenced *D*. *antarctica* from the Weddell Sea and compare it to specimens from the Ross Sea. The aims of this study are thus threefold: (1) to improve species delimitation of the two Antarctic *Doto* species; (2) to provide a basis for anatomical comparison of the species of *Doto*; and (3) to disentangle the phylogenetic conundrum of *Doto* species. We also provide an evolutionary scenario of the changes in *Doto* anatomy for all the species for which molecular data are available.

## Material and Methods

### Sample collection

Nudibranch samples were collected in the eastern Weddell Sea (Antarctica) during the ANT XV/3 cruise (1998) [27], and during the ANT XXI/2 cruise (2003–2004) of the R/V Polarstern (Alfred Wegener Institute, Bremerhaven, Germany). Several specimens of *D*. *antarctica* and egg masses were collected at depths ranging from 65 to 433 m at several stations (see Table 1). For *D*. *carinova* Moles, Avila & Wägele n. sp., only one specimen and its egg masses were collected from station code PS65/276-1. Samples were photographed alive, anaesthetised with 10% magnesium chloride for 1h, and then transferred to 70% ethanol for morphological analysis. One specimen of *D*. *antarctica* was preserved in 10% formaldehyde/sea water for histology (PS65/166-1), and two specimens were frozen and then transferred to 100% ethanol for sequencing (48/033). Additional *Doto* species were also collected from the Mediterranean Sea and sequenced to increase the number of taxa from the ones already available in GenBank (see S1 Table). Antarctic samples were collected with the permission of the Spanish Polar Committee (CPE; www.idi.mineco.gob.es/portal/site/MICINN). Mediterranean samples were collected under the permission issued by the Catalan Government (www.gencat.cat/darp).

**Table 1:** Sampling stations where *D*. *antarctica* and *D*. *carinova* Moles, Avila & Wägele n. sp. were collected. Kapp Norvegia is situated in the eastern Weddell Sea.

Specimens (n°)	Cruise	Date	Area	Station code	Operation	Latitude (S)	Longitude (W)	Depth (m)
*D*. *antarctica* (12) *+* egg masses (8)	ANTXV/3	29/01/98	North of Kapp Norvegia	48/033	TV grab	71° 7.3’ S	11° 28.3’ W	65
*D*. *antarctica* (1)	ANTXV/3	29/01/98	North of Kapp Norvegia	48/037	Dredge	71° 6.7’ S	11° 28’ W	146
*D*. *antarctica* (1)	ANTXV/3	01/02/98	North of Kapp Norvegia	48/071	Bottom trawl	71° 50.5’ S	10° 32.8’ W	231
Egg masses *D*. *antarctica* (3)	ANTXV/3	02/02/98	North of Kapp Norvegia	48/077	Agassiz trawl	71° 8.6’ S	12° 26.6’ W	433
*D*. *antarctica* (1)	ANTXV/3	16/02/98	Kapp Norvegia	48/198	Dredge	71° 17’ S	12° 36.6’ W	416
*D*. *antarctica* (1)	ANTXV/3	18/02/98	Kapp Norvegia	48/214	Dredge	71° 7.2’ S	11° 28.8’ W	110
Egg masses *D*. *antarctica* (6)	ANTXV/3	27/02/98	Kapp Norvegia	48/277	Agassiz trawl	71° 18.2’ S	12° 16.4’ W	177
*D*. *antarctica* (1)	ANTXXI/2	25/11/03	Bouvet Island	PS65/029-1	Agassiz trawl	54° 31.59’ S	3° 13.05’ E	377
*D*. *antarctica* (1)	ANTXXI/2	15/12/03	Eastern Weddell Sea	PS65/166-1	Bottom trawl	70° 56.83’ S	10° 32.61’ W	338
*D*. *antarctica* (1)	ANTXXI/2	29/12/03	Eastern Weddell Sea	PS65/280-1	Agassiz trawl	71° 7.15’ S	11° 26.23’ W	228
*Doto carinova* n. sp. (1) + egg masses (4)	ANTXXI/2	28/12/03	Eastern Weddell Sea	PS65/276-1	Agassiz trawl	71° 6.44’ S	11° 27.76’ W	277

### Morphological analysis

The buccal mass of *D*. *antarctica* (48/033) was immersed in potassium hydroxide for up to three hours to dissolve the organic tissues and then rinsed with distilled water. The radula was mounted on metallic stubs with bioadhesive carbon sticky tabs and coated with carbon for scanning electron microscopy (SEM). One specimen of *D*. *antarctica* (PS65/166-1), an egg mass (48/033), and the egg mass of *D*. *carinova* n. sp. (PS65/276-1) were dehydrated in an ethanol series and embedded in HEMA for histological analysis (Kulzer’s method; see [28]). Serial sections (2.5 μm thick) were stained with Toluidine blue, which specifically stains acid mucopolysaccharides red to violet, and neutral mucopolysaccharides and nucleic acids, as well as proteins in various shades of blue.

**Fig 1:**
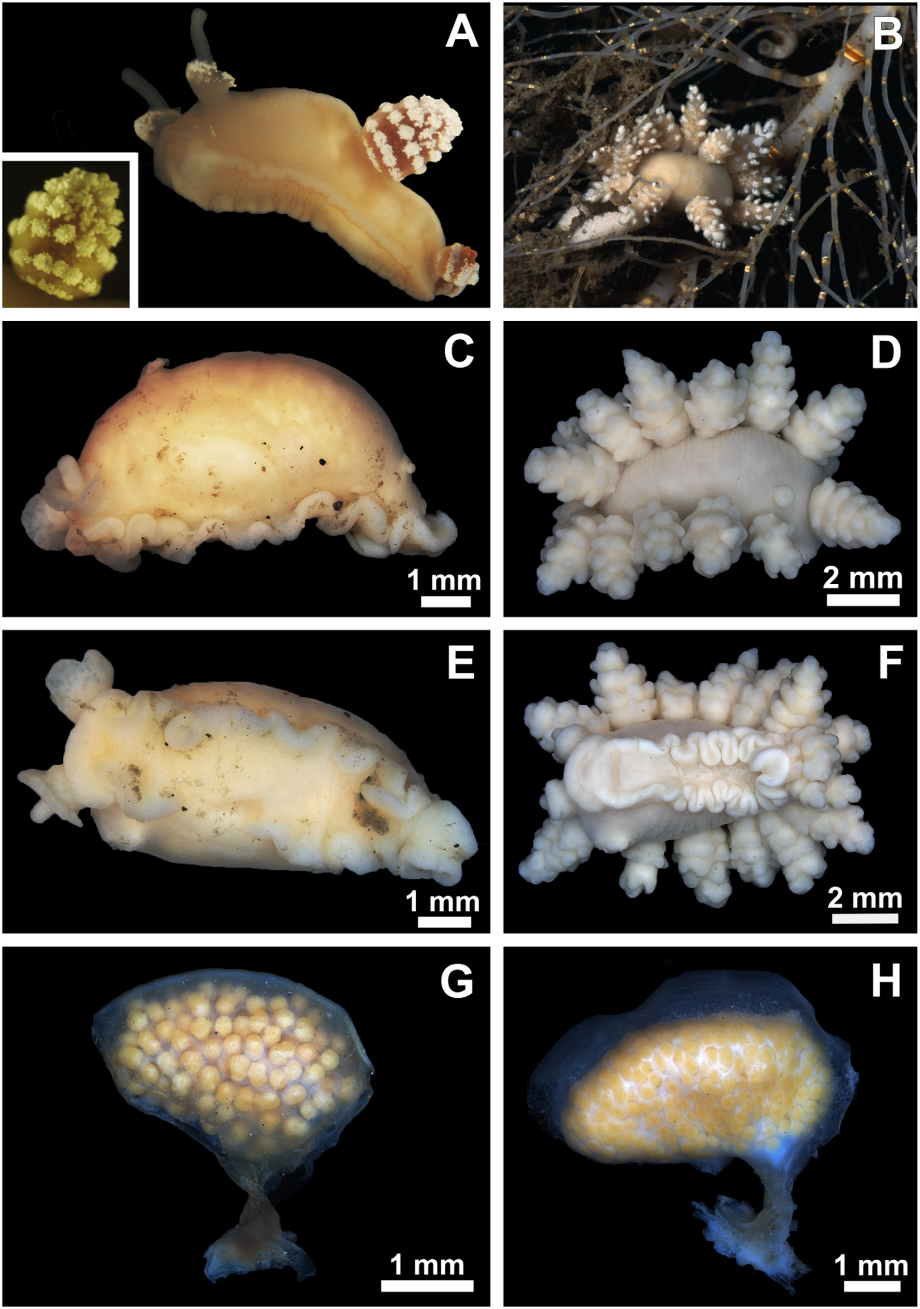
Photographs of *D*. *antarctica* (left column: A,C,E,G) and *D*. *carinova* Moles, Avila & Wägele n. sp. (right column: B,D,F,H); specimens subjected to micro-CT reconstruction. **A** Live animal, where most of the cerata were lost; close up of the cerata. **B** Live picture right after collection, showing the *D*. *carinova* n. sp. spawning on top of the gorgonian *Primnoisis antarctica* (Isididae). **C**–**D** Lateral and dorsal view of the preserved animals. **E**–**F** Ventral view of the preserved animals. **G**–**H** Lateral view of the preserved egg masses.

For micro-CT analysis, *D*. *antarctica* (PS65/280-1) and *D*. *carinova* n. sp. (PS65/276-1) specimens (Fig 1) were dehydrated in a graded series of ethanol, contrasted with 1% iodine metal (I_2_) dissolved in 100% ethanol (I2E) for 24 h, and transferred to 100% ethanol. Subsequently, each specimen was mounted on a pipette tip containing 100% ethanol, with the specimen arrested, later mounted on a pin with superglue. The microscopic X-ray tomography scan was performed with an XRadia Micro XCT-200 (Carl Zeiss X-ray Microscopy Inc.) using the 4x object lens unit, at 40 kV and 200 μA, with a pixel size of 5.77 and 4.98 μm for *D*. *antarctica* and *D*. *carinova* n. sp., respectively. Tomography projections were then reconstructed using the software provided by XRadia. For image segmentation the software platform Amira^®^ 5.4. (FEI, Visualization Science Group) was used. Images from micro-CT scans were compared with histological sections (2.5 μm thick) for reciprocal illumination. A graphical 3D PDF reconstruction of both species was performed using Deep Exploration. 3D PDFs can be opened in Adobe Acrobat Reader and activated by clicking on it (see S1 3D PDF and S2 3D PDF).

### Nomenclatural acts

The electronic edition of this article conforms to the requirements of the amended International Code of Zoological Nomenclature, and hence the new names contained herein are available under that Code from the electronic edition of this article. This published work and the nomenclatural acts it contains have been registered in ZooBank, the online registration system for the ICZN. The ZooBank LSIDs (Life Science Identifiers) can be resolved and the associated information viewed through any standard web browser by appending the LSID to the prefix “http://zoobank.org/”. The LSID for this publication is: urn:lsid:zoobank.org:pub:13848907-E389-4C2F-93D8-A1238DA1B853. The electronic edition of this work was published in a journal with an ISSN, and has been archived and is available from the following digital repositories: PubMed Central, LOCKSS.

### DNA amplification

We sequenced two specimens of *D*. *antarctica* (Station code 48/033; see Table 1), but we were not able to sequence *D*. *carinova* n. sp. due to inadequate chemical fixation for molecular analyses. Total genomic DNA was extracted from small pieces of foot tissue using DNeasy Tissue Kit (Qiagen, Valencia, CA, USA). Molecular markers included two fragments of the mitochondrial genes cytochrome *c* oxidase subunit I (COI) and 16S rRNA, and the nuclear gene histone H3. A fragment of about 593 bp of the mitochondrial protein-encoding gene COI was amplified using the primers LCO1490 and HCO2198 [29]. A fragment of about 383 bp of the 16S rRNA gene was amplified using the primer pair 16Sar-L and 16Sbr-H [30]. A fragment of about 310 bp of the protein-encoding gene histone H3 was amplified using the primer pair H3AD5’3’ and H3BD5’3’ [31]. PCR amplifications were carried out in a 10 μL-reaction volume including 5.1 μL of Sigma dH_2_O, 3.3 μL REDExtract-N-Amp^™^ PCR ReadyMix (Sigma Aldrich, St. Louis, MO, USA), 0.3 μL of each primer, and 1 μL of genomic DNA. Polymerase chain reaction (PCR) programs for COI and 16S rRNA involve an initial denaturing step (94°C for 5 min) followed 40 cycles of denaturation (94°C for 30 s), annealing (44–50°C for 30 s), and extension (72°C for 30 s), with a final extension step at 72°C for 5 min. For histone H3, the initial denaturation step was conducted at 94°C for 3 min followed by 35 cycles including denaturation at 94°C for 35 s, annealing at 50°C for 1 min, and extension at 72°C for 15 s, with a final extension step at 72°C for 2 min. Amplified products were purified using microCLEAN (Microzone Ltd., Sussex, UK) and sequenced at the UB Scientific and Technological Centers (CCiT-UB) on an ABI 3730XL DNA Analyzer (Applied Biosystems).

### Phylogenetic analysis

Chromatograms were visualized and sequences were assembled in Geneious Pro 8.1.5 [32]. These were compared against the GenBank nucleotide database with the BLAST algorithm [33] to check for contamination. Alignments were trimmed to a position at which more than 50% of the sequences had nucleotides and missing positions at the ends were coded as missing data. All new sequences have been deposited in GenBank (see S1 Table for accession numbers). We used GBlocks 0.91b on the final trimmed alignment for identifying and excluding blocks of ambiguous data in single, non-codifying gene alignments (16S) with relaxed settings [34].

Bayesian inference (BI) and maximum likelihood (ML) analyses were conducted on the concatenated alignment of the three genes. BI analyses were performed using MrBayes ver. 3.2.5 [35] with a unique GTR model of sequence evolution [36] with corrections for a discrete gamma distribution and a proportion of invariant sites (GTR + Γ + I) [37] specified for each partition, as selected in jModelTest ver. 2.1.7 [38] under the Akaike Information Criterion [39]. Two runs, each with three hot chains and one cold chain, were conducted in MrBayes for 20 million generations, sampling every 2000th generation, using random starting trees. The analysis was performed twice, and 25% of the runs were discarded as burn-in after checking for stationarity with Tracer v.1.6 [40]. The remaining trees were combined to find the maximum *a posteriori* probability estimate of phylogeny.

ML analyses were conducted using RAxML ver. 8.1.2 [41]. For the maximum likelihood searches, a unique GTR model of sequence evolution with corrections for a discrete gamma distribution (GTR + Γ) [37] was specified for each data partition, and 500 independent searches were conducted. Nodal support was estimated via the rapid bootstrap algorithm (1000 replicates) using the GTR-CAT model [42]. Bootstrap resampling frequencies were thereafter mapped onto the optimal tree from the independent searches.

## Results

### Systematics

Cladobranchia Willan & Morton, 1984

Dotidae Gray, 1853

*Doto* Oken, 1815

Type species: *Doris coronata* Gmelin, 1791

#### *Doto antarctica* Eliot, 1907

**Fig 2:**
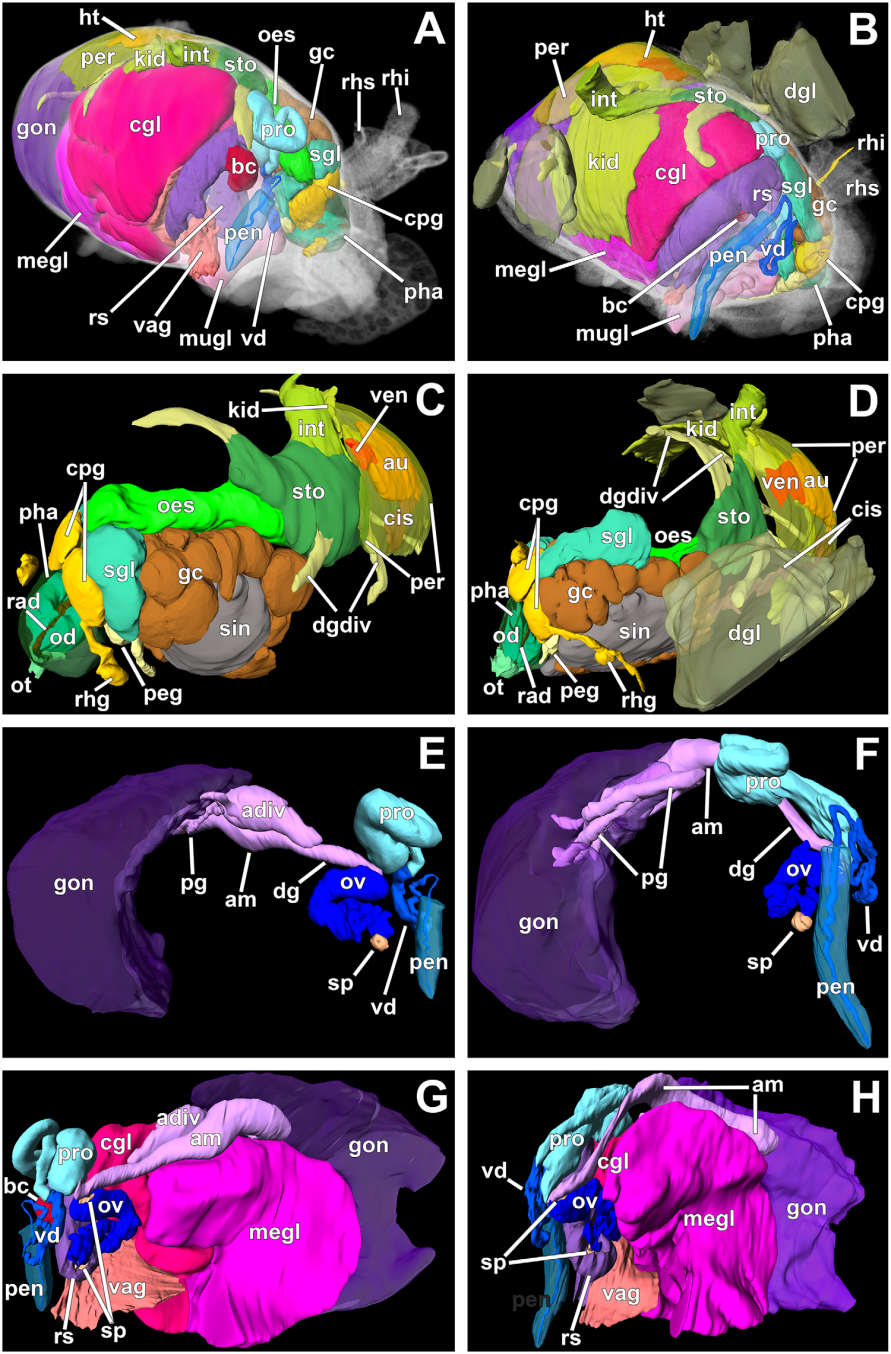
Micro-CT reconstructions of the internal organs of *D*. *antarctica* (left column) and *D*. *carinova* Moles, Avila & Wägele n. sp. (right column). **A**–**B** Right antero-lateral view of all reconstructed organs. **C**–**D** Left antero-lateral view of the circulatory, digestive, excretory, and nervous systems. **E**–**F** Right lateral view of the male reproductive system. **G**–**H** Left lateral view of the reproductive system (mucus gland is not depicted here since it covers the whole view). *am* ampulla; *adiv* ampulla diverticulum; *au* auricle; *bc* bursa copulatrix; *cis* circulatory sinuses; *cgl* capsule gland; *cpg* cerebropleural ganglion; *dg* distal gonoduct; *dgdiv* digestive gland diverticula; *dgl* digestive gland (only depicted in *D*. *carinova* n. sp.); *gc* giant cells; *gon* gonad; *ht* heart; *int* intestine; *kid* kidney; *megl* membrane gland; *mugl* mucus gland; *oes* oesophagus; *od* odontophore; *ot* oral tube; *ov* oviduct; *peg* pedal ganglion; *pen* penis; *per* pericardium; *pg* proximal gonoduct; *pha* pharynx; *pro* prostate; *rad* radula; *rhg* rhinophoral ganglion; *rhi* rhinophore; *rhs* rhinophoral sheath; *sgl* salivary gland; *sin* sinus; *sp* sphincter; *sto* stomach; *vag* vagina; *vd* vas deferens; *ven* ventricle.

**Fig 3:**
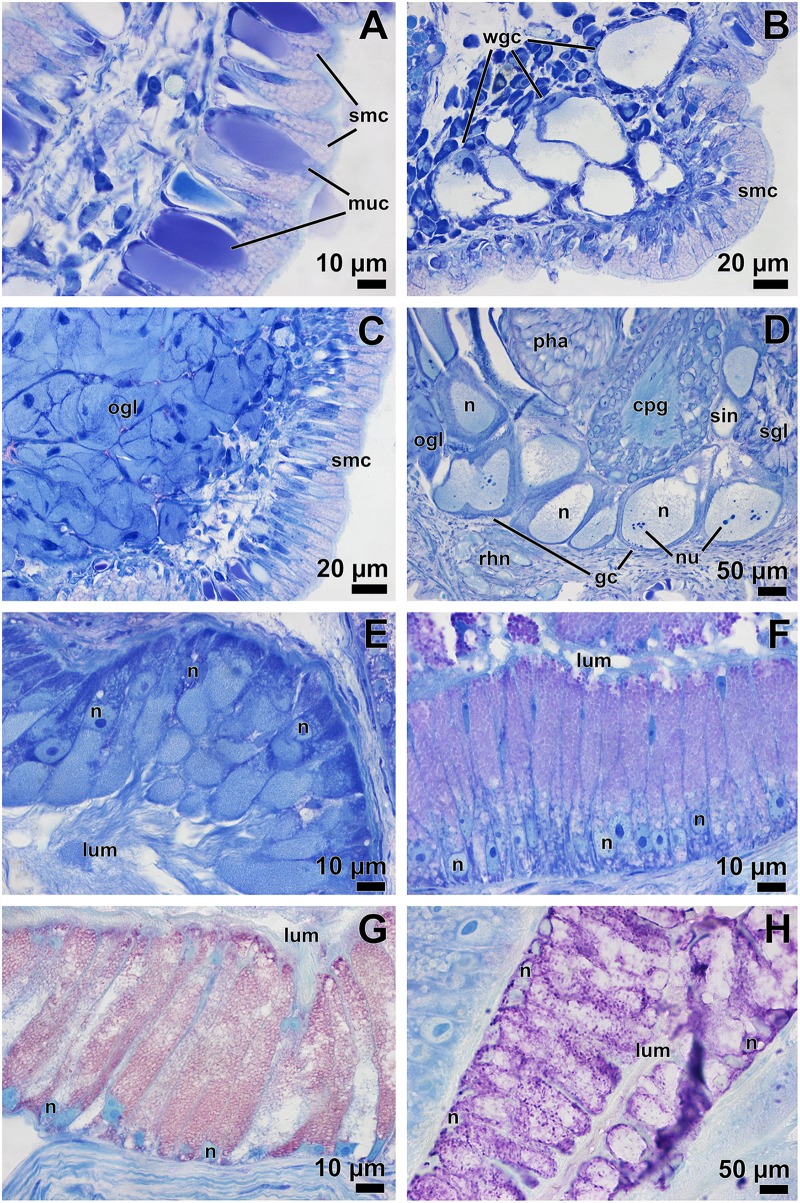
Histological slides of the glandular structures in *D*. *antarctica*. **A** Epidermis of the rhinophoral sheath. **B** Detail of one cerata tubercle showing defensive glandular cells (white punctuation in live animals). **C** Oral glands. **D** Giant neurones chain attached to the cerebropleural ganglion; a thin cortex, large nucleus, and nucleolus of each cell can be seen. **E** Detail of the prostate glandular cells. **F** Detail of the capsule glandular cells. **G** Detail of the membrane glandular cells. **H** Detail of the glandular mucus cells. *gc* giant cells; *lum* lumen; *muc* glandular mucus cell; *n* nucleus; *nu* nucleolus; *ogl* oral glands; *pha* pharynx; *rhn* rhinophoral nerve; *sin* sinus vessel; *sgl* salivary gland; *smc* specialised multivacuolised cell; *wgc* white glandular cells.

**Fig 4:**
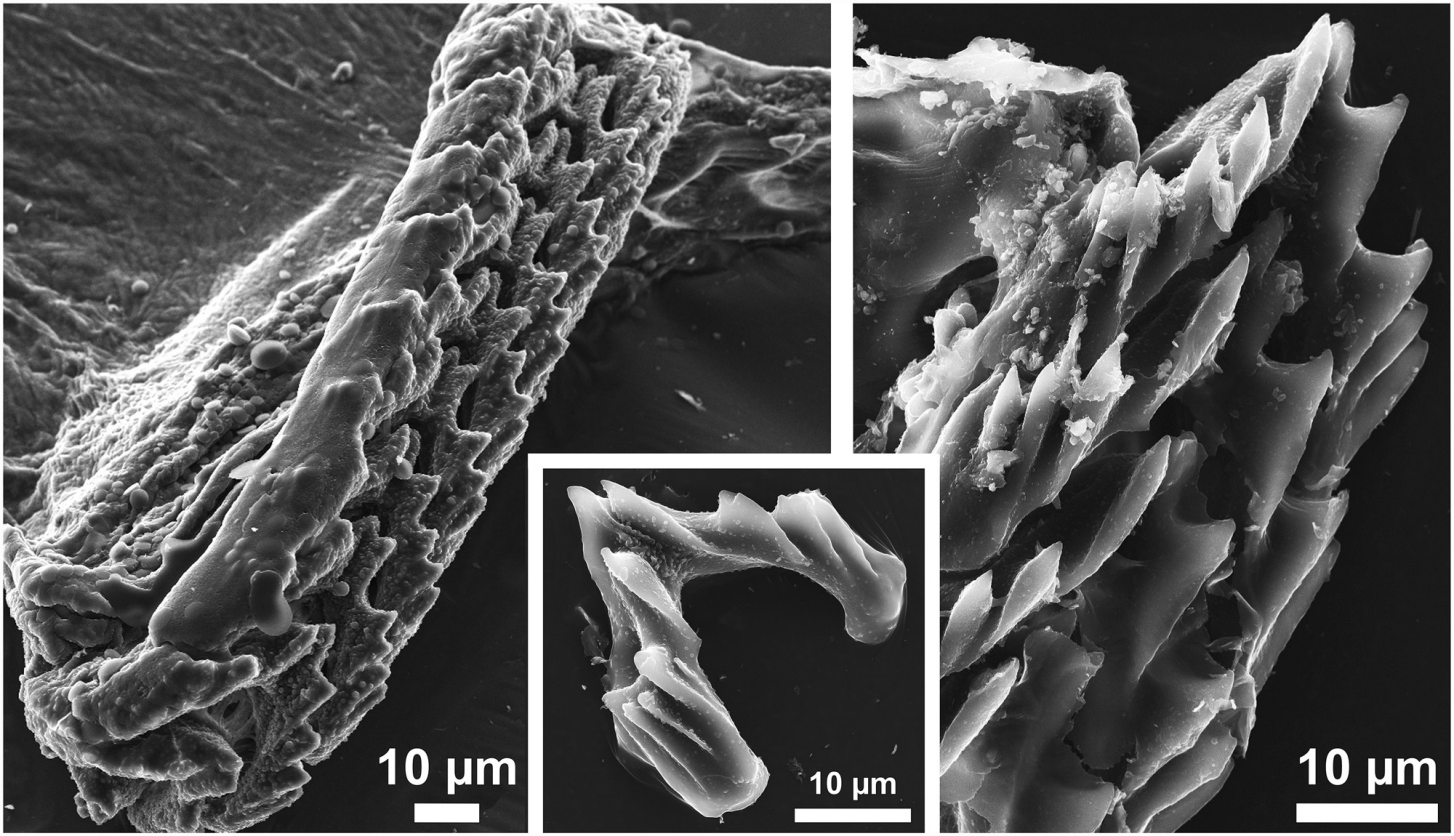
Scanning electron micrographs (SEM) of the radular teeth of *D*. *antarctica*.

(Figs 1–4) (See S1 3D PDF of the reconstructed anatomy of the anterior region of the specimen)

***Material examined*:** See Table 1. Deposited in SNSB Zoologische Staatssammlung München (Catalog number ZSM Moll 2016115).

***Distribution*:** Ross and Weddell Seas, King George Island (South Shetland Islands), and Bouvet Island.

***External morphology*:** (Fig 1A, 1C and 1E). Body short and bulged dorsally mostly due to reproductive system; young and adult specimens measured 4–9 x 2–5 x 2–3.5 mm (length:width:height). Body and cerata pale brown, intensified in cerata; containing bright white spots (corresponding to huge glandular cells) on tip of rhinophores, edge of rhinophoral sheaths, and each tubercle on cerata. Velum broad and rounded. Tentacular processes absent. Rhinophores transparent, thin, smooth, blunt; rhinophoral sheath 1/3 of rhinophore size, elongated in frontal side (giving resemblance of “calla lily” inflorescence). Six cerata pairs, short, rounded, 2–3 mm high, progressively smaller towards tail, connected to body by narrow connection; 4–5 circlets containing up to 12 short tubercles (largest circlet) close to each other, apex of cerata of same shape. Pseudobranchs absent. Cerata in live specimens easily autotomized upon manipulation, discharging white content of glandular cells. Anal papilla large, placed dorsally in mid-right position. Genital apertures below and in between 1^st^ and 2^nd^ cerata on right side. Foot narrow, linear, rounded anteriorly, tapering posteriorly to short, blunt tail.

***Digestive system*:** (Fig 2A and 2C). Mouth opening ventrally between oral veil and foot; oral tube relatively short and surrounded by follicular, blue-staining, oral glands (Fig 3C). Jaws thin, membranous, without any appreciable ornamentation. Pharynx bulbous, inner lining presenting thin cuticle; posteriorly projected downwards due to odontophore and upwards (suctorial pump). Radular formula 72 x 0.1.0; rachidian arched, pointed tip; bearing five denticles along border, unequal among and within teeth (see Fig 4). Paired, saccular salivary glands leading dorso-posteriorly to pharynx by short duct; attached to posterior part of cerebropleural ganglia; composed by huge granular cells containing high amount of secretory vacuoles (see Fig 3D). Oesophagus opening at upper end of pharynx; widening, connecting to stomach right before location of anal papilla. Stomach widening, becoming flattened; from there, several digestive glandular diverticula reaching into cerata; from anterior part of stomach, two digestive gland ducts reaching into left and right cerata; from posterior part of stomach, one large digestive gland duct opening, composed of several diverticula reaching posterior cerata. Posterior branch and diverticula covered by gonad. Digestive gland containing typical digestive epithelium only present in cerata, here forming rather diffuse, racemose tissue (not depicted in Fig 2). Intestine short, thick, leading through densely ciliated anal papilla to outside.

***Reproductive system*:** (Fig 2E and 2G). Diaulic. Ovotestis occupying whole posterior body region, reaching dorsally far until mid-longitudinal section. Ampulla convoluted; gonoducts connecting to small, globose, bean-shaped ampulla diverticulum placed in proximal part, ending blind; lying dorsally between gonad, mucus and membrane glands; proximally to diverticulum, elongated ampulla narrowing to distal gonoduct, branching into vas deferens and oviduct. Proximal vas deferens widening into prostate, the latter forming a loop in dorso-anterior region of animal, becoming thinner; composed by elongate cells, containing basal nucleus, cytoplasm filled of small, blue-staining granules (Fig 3E). Distal vas deferens after prostatic part decreasing in diameter, connecting to penis. Penis unarmed, conical, relatively short; placed in ovoid penial sheath, which can be sometimes seen through penial pore in preserved specimens. Male and female genital openings in antero-lateral right position.

Oviduct starting with a sphincter, widening, entering distally the vaginal duct, which widens directly into receptaculum seminis (flow through system). This connection separated by a distinct sphincter. At same area oviduct entering nidamental glands.

Receptaculum seminis wide, highly elongated, folded, leading to vagina distally. Bursa copulatrix small, rounded, saccular; placed at middle part of vaginal duct/receptaculum seminis. Vagina flattened, highly ciliated, leading outside by wide aperture; sharing wide atrium with mucus gland. We follow the functional terminology of Klussmann-Kolb [43] for nidamental glands, composed of capsule gland, followed by membrane gland, leading into mucus gland. Capsule gland occupying right antero-lateral region of animal; composed by thin, columnar cells containing small microvilli in apical pole, basal nucleus, cytoplasm entirely composed of bluish granules, which become increasingly pinkish towards end of gland (Fig 3F). Membrane gland lying ventrally under capsule gland, extending further posteriorly under gonad, composed by columnar cells filled with reddish granules (Fig 3G). Mucus gland, the largest part of the nidamental gland, occupying ventrally about 2/3 of body, also extending anteriorly; composed of columnar cells containing basal nucleus, many small, ovoid, violet granules (Fig 3H).

***Nervous system*:** (Fig 2C). Oesophageal nerve ring composed of four ganglia. Cerebral ganglia fused to pleural ganglia into cerebropleural ganglia. Ganglia composed by cortical layer of neurones encircling central neuropil. From neuropil of cerebropleural ganglion one nerve connects to small rhinophoral ganglion, placed right at basal part of each rhinophore. Rhinophoral nerve short leading to top of rhinophore; short optic nerves connecting to eyes. Eyes containing large, spherical lens; retina showing melanin granules. Both cerebropleural ganglia nearly close together, no cerebral commissure visible. Pedal ganglia, interconnected by relatively long commissure, lying close to cerebropleural ganglia with short connectives. From these, one nerve running anteriorly, another down to foot passing through giant cells (GCs) and sinus. A total of 27 GCs measuring 168.7 ± 20.14 μm (mean ± sd) in maximum diameter, seen close to left cerebropleural and pedal ganglia and right salivary gland, extending posteriorly forming a circle in left anterior region of animal; only one GC lying under left pedal ganglion; cytoplasm remains mostly occupied by huge nucleus containing faint bluish fibrillar appearance (appearing shining in micro-CT like the cortical neurons of ganglia); several amoeboid-shaped nucleoli, staining dark blue, apparent (Fig 3D). All GCs contact to each other, close to central hemolymphatic sinus and/or to small vessel-like sinus. The whole GC complex occupying one third of anterior body volume.

***Circulatory and excretory systems*:** (Fig 2C). Pericardium wide, flattened; situated dorsally, behind anal papilla. Auricle connecting to small anterior lying ventricle. Vessel-like structure running from auricle anteriorly to edge of pericardium, reaching cerata. Kidney flattened, connecting ventrally on right side to pericardium; extending far into posterior part of animal, widening notably; nephroduct extending far into anal papilla, leading outside close to anus.

***Epithelial glandular structures*:** Notal epithelium formed by unicellular layer of pinkish multivacuolized cells (specialised vacuolated epithelium), interspersed with mucus glandular cells (Fig 3A). Multivacuolized cells prismatic in shape, having basal nucleus, presenting microvilli all over apical part. Mucus gland cells presenting one huge vacuole occupying whole cytoplasm, containing different shades of homogeneous violet-stained contents (acid mucopolysaccharides); ubiquitously seen in notum, rhinophores, and cerata. Subepithelial clusters of large, bluish, glandular cells observed in rhinophore tips, sheath, and cerata tubercles; probably responsible for whitish appearance in live animals; probably exuded their contents when animal disturbed, thus defensive function is proposed (Fig 3B).

***Egg mass*:** (Fig 1G). Oval, slightly bean-shaped, 3.5–4 x 3.5 x 2 mm (length:heigth:width); enveloped by thick membrane composed of four layers. Outermost layer with maximal width of 3.3 μm measured, blue coloured; following outer layer 53.3 μm, pink, containing profuse wholes; inner layer 40.51 μm, pink; innermost 48.39 μm, purple. Egg mass presenting dorsal keel, of around 0.4 mm, extending far from one side to the other. Whole egg mass attached to substrate by twisted stalk placed in mid-lateral position. Dorsal keel and stalk composed mainly by first three layers. Egg capsules measured 336.95 ± 32.16 μm (mean ± sd); cytoplasm mainly composed by blue-staining protein platelets.

***Ecology*:** The 19 specimens were found in benthic ecosystems at 65–500 m depth. Some specimens were found laying egg masses on two unidentified hydrozoans of the genera *Oswaldella* Stechow, 1919 (Plumularioidea: Kirchenpaueriidae) and *Antarctoscyphus* Peña Cantero, Garcia Carrascosa & Vervoort, 1997 (Sertulariidae).

#### *Doto carinova* Moles, Avila & Wägele n. sp

**Fig 5:**
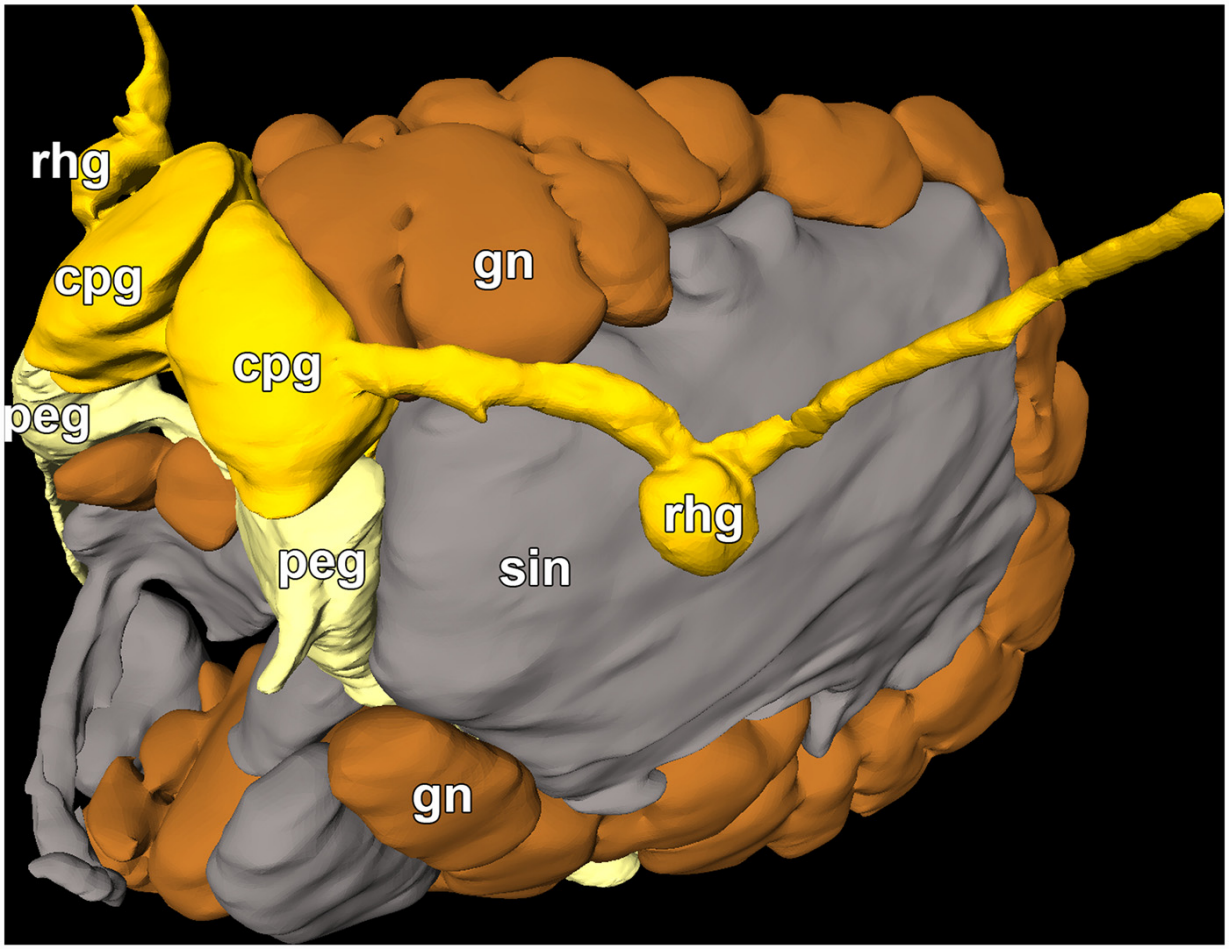
Left antero-lateral view of the micro-CT reconstruction of the nervous system of *D*. *carinova* Moles, Avila & Wägele n. sp. *cpg* cerebropleural ganglion; *gc* giant cells; *peg* pedal ganglion; *rhg* rhinophoral ganglion; *sin* sinus.

**Fig 6:**
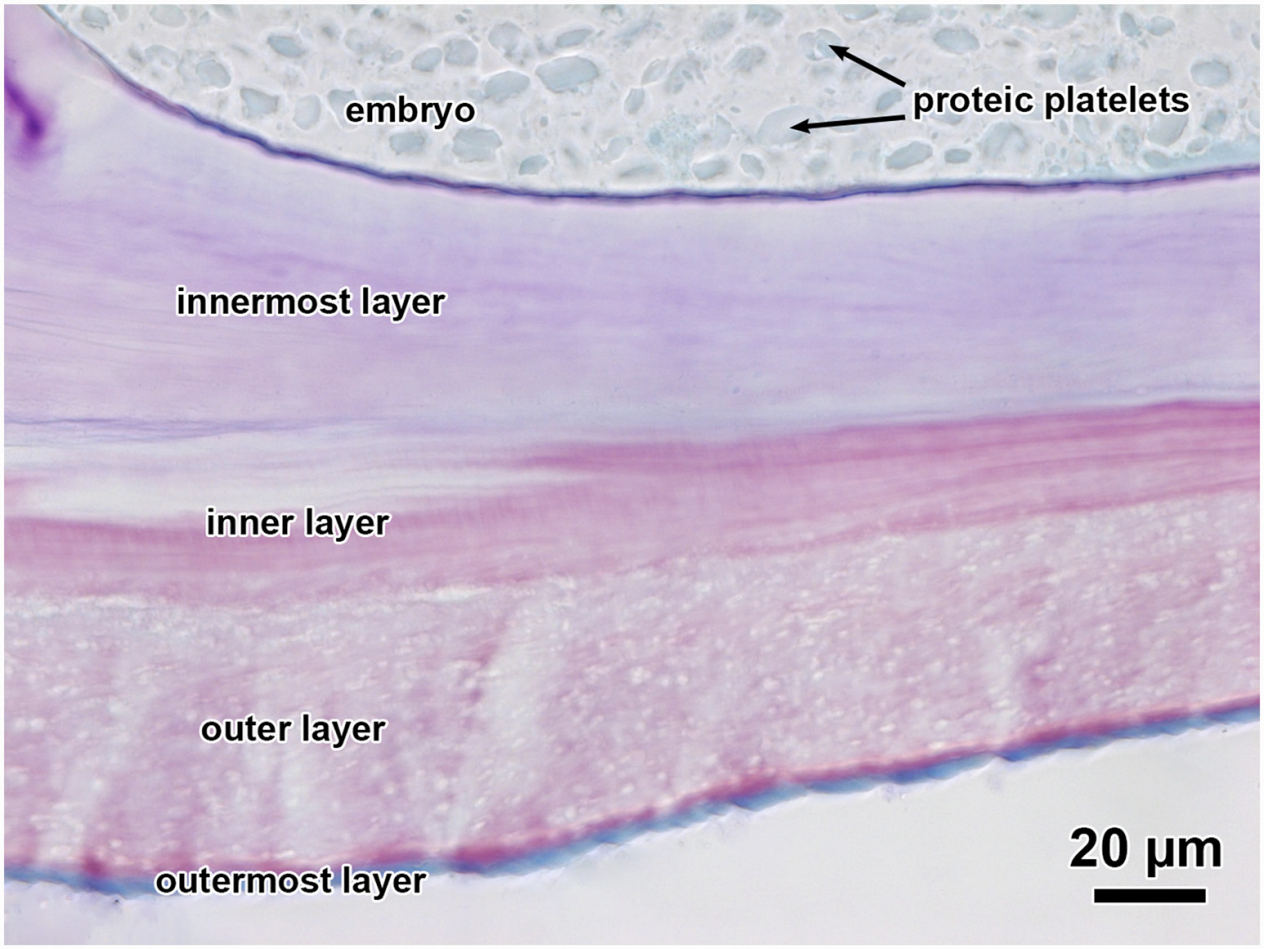
Histological slide of outer surface of the egg mass in *Doto carinova* Moles, Avila & Wägele n. sp.

(Figs 1, 2, 5 and 6) (See S2 3D PDF of the reconstructed anatomy of the anterior region of the specimen)

http://zoobank.org/NomenclaturalActs/7023623D-B021-4D83-B216-B8C6ACDCDD19

***Type locality*:** Eastern Weddell Sea (71° 6.44’ S; 11° 27.76’ W), 277 m depth.

***Material studied*:** A single specimen collected during the Antarctic cruise ANT XXI/2 (see Table 1). Deposited in SNSB Zoologische Staatssammlung München (Catalog number ZSM Moll 2016114).

***External morphology*:** (Fig 1B, 1D and 1F). Body bulged anteriorly due to reproductive system; live specimen measured 13 mm in length, 10 x 4 x 5 mm (length:width:height) when preserved. Body and cerata creamy coloured; presenting bright white spots (glandular cells) in tip of rhinophores, along edge of rhinophoral sheath, and each tubercle in cerata. Velum short, rounded. Oral tentacular processes absent. Eight long, pointed cerata pairs, 6 mm high, narrow connections to body; apex long, lobulated; elongate tubercles on cerata disposed in 4–5 circlets with maximum of nine per circlet. Pseudobranchs absent. Rhinophores transparent, thin, smooth, blunt; rhinophoral sheath short, cylindrical, narrow (calyciform), slightly expanded anteriorly, being up to 1/2 of rhinophore size. Anal papilla large, placed dorsally in mid-right position. Genital apertures lying under right row of cerata, between 1^st^ and 2^nd^. Foot narrow, rounded anteriorly, tapering posteriorly to short, blunt tail.

***Digestive system*:** (Fig 2B and 2D). Mouth opening ventrally between oral veil and foot; oral tube relatively short, surrounded by follicular oral glands. Pharynx bulbous, containing thin cuticle. Radula not analysed since the specimen was not dissected. Paired salivary glands leading to middle part of pharynx, entering via long ducts; right one stretching along posterior part of cerebropleural ganglion, not reaching backwards as left one, which extends far posteriorly along oesophagus; both being long, saccular, widening progressively posteriorly (giving pyriform appearance). Oesophagus opening at posterior dorsal side of pharynx; widening, connecting to stomach. Stomach widening, flattening, opening into two digestive gland diverticula, which run into anterior cerata; third (posterior) branch of digestive gland opening posteriorly from stomach, running to posterior part of animal, branching off diverticula which reach into last cerata pairs. Digestive gland diverticula connecting to typical diffuse, racemose digestive gland, situated exclusively in cerata, occupying it almost entirely. Intestine cylindrical in cross section, opening from posterior part of stomach, leading outside by long anal papilla.

***Reproductive system*:** (Fig 2F). Diaulic. Gonad occupying whole posterior body region, reaching far until mid-longitudinal section on dorsal side. Smaller gonoducts uniting into anterior gonoduct, leading into large, elongated ampulla; the latter lying dorsally underneath gonad, close to mucus and membrane glands. From distal part of ampulla thin distal gonoduct extending anteriorly to prostate and oviduct. Prostate long, isodiametric, folded, leading to thin, contorted distal vas deferens, connecting to penis. Penis long, conical, unarmed; placed in ovoid penial sheath; tip of penis seen outside penial pore in preserved specimen. Genital openings lying in antero-right position.

Oviduct starting with a sphincter, widening, branching into distal oviduct, leading directly into nidamental glands and vaginal duct, opening directly into receptaculum seminis (flow through system). Bursa copulatrix attached to widened part of receptaculum seminis. Both placed close to and below prostate; oviduct separated from bursa copulatrix again by additional sphincter. Bursa copulatrix wide, highly elongated, folded, leading distally to vagina. Receptaculum seminis small, rounded, saccular; attached to vaginal canal in middle position. Vagina short, flattened, leading outside by wide aperture; sharing wide atrium with nidamental glands.

Nidamental glands composed of: 1) capsule gland occupying right antero-lateral region, 2) membrane gland placed right under capsule gland, extending further posteriorly below gonad, 3) highly folded mucus gland reaching ventrally far into anterior and posterior part of body.

***Nervous system*:** (Fig 5). Oesophageal nerve ring composed of four ganglia. Cerebral ganglia fused to pleural ganglia into cerebropleural ganglia; standing close without distinct commissure; also standing close to pedal ganglia, the latter interconnected by relatively long commissure. Each cerebropleural ganglion sending one nerve to small rhinophoral ganglion, placed right at bottom of each rhinophore; from there, a short nerve leading to top of rhinophore. From pedal ganglia, one nerve running down to foot, through giant cells (GCs) and sinus. Forty interconnected GCs can be seen (appearing shining in micro-CT like the cortical neurons of ganglia), some close to cerebropleural and pedal ganglia and right salivary gland; extending posteriorly, forming a circle in left anterior region of animal body; only two GCs found under pedal ganglia; all GCs attached to large hemolymphatic sinus; whole GCs complex occupying one third of body volume in anterior part.

***Circulatory and excretory systems*:** (Fig 2D). Pericardium wide, flattened, occupying dorsal part of body right behind anal papilla. Heart composed by large auricle and anterior lying ventricle, both placed in longitudinal axis. Several sinuses connecting to pericardium, eventually to auricle, receiving oxygenated hemolymph from each ceras; running close to digestive gland diverticula (only two at left and one at right side could be seen and depicted in micro-CT reconstruction). Kidney placed ventrally of pericardium; directly connected in anterior right position through small duct; nephroduct leading to anal papilla; nephropore close to anus.

***Egg mass*:** (Fig 1H). Reniform, bean-shaped egg masses measured 5.5–8.4 x 3.5–6 x 2–2.5 mm (length:heigth:width); slightly asymmetrical in transverse section, being bulged in one side; enveloped by thick membrane, composed by four layers. Outermost layer staining blue; 3 μm in width; following outer layer 33.3 μm, pink, containing abundant wholes; inner layer 23.71 μm, pink; innermost 27.7 μm, purple (Fig 6); dorsal keel and stalk only composed by the three outer layers. Dorsal keel measuring 0.8–1 mm in length by 118 μm in width at wider base, running from one side to the other. Whole egg mass attached to substrate by one twisted stalk placed in mid-lateral position. Live egg capsules white, becoming orange when preserved, measuring 357.48 ± 29.8716 μm (mean ± sd); cytoplasm mainly composed by blue-staining protein platelets.

***Ecology*:** *Doto carinova* n. sp. was collected from muddy bottoms at 277 m depth in a benthic community with abundant gorgonians (*Thouarella*, Primnoidae), sponges, colonial tunicates, hydrozoans, bryozoans, amphipods, ophiuroids, and molluscs. The animal was found laying an egg mass on the Isididae gorgonian *Primnoisis antarctica* (Studer, 1878), close to other three additional egg masses.

***Etymology*:** In the name *Doto carinova* n. sp., the specific epithet is an apposition derived from the words *carina* (= keel) and *ova* (= eggs) in Latin, referring to the pronounced keel observed in the egg mass.

***Remarks*:** (Table 2). Externally *D*. *carinova* n. sp. differs from the sympatric *D*. *antarctica* in having a longer and paler body, a shorter velum, eight (versus six) longer cerata with less and more pronounced tubercles, a short and circular rhinophoral sheath, and a slightly larger anal papilla. The salivary glands differ notably from these of *D*. *antarctica*, by being longer, presenting longer salivary ducts, and presenting the right salivary gland reaching far posteriorly. The ampulla is folded and presents a proximal bean-shaped diverticulum in *D*. *antarctica*, while it is elongated and isodiametric in *D*. *carinova* n. sp. Moreover, the prostate is longer and folded in *D*. *carinova* n. sp., while in *D*. *antarctica* it is short, wide proximally, and forms a pronounced loop. The penis is also longer in the new species. More GCs (40) can be seen in the new species than in *D*. *antarctica* (27).

**Table 2:** Differential characters among *D*. *carinova* Moles, Avila & Wägele n. sp. and *D*. *antarctica*.

	*Doto carinova* n. sp.	*Doto antarctica* Eliot, 1907
**External morphology**		
colour	yellowish, creamy, pale	brownish
rhinophoral sheath	rounded, calyciform	elongated anteriorly, “calla lilly”-shaped
velum	narrow	broad
cerata	8	6
tubercles	up to 9, long (6 mm), lobulated	up to 12, short (3–4 mm), rounded
**Digestive system**		
salivary glands	pyriform, elongated, right extending back over oesophagus	saccular, roundish, not extending back
**Nervous system related**		
giant cells	40	27
**Reproductive system**		
ampulla	elongated	convoluted; round, bean-shaped proximal diverticulum, elongated distal part
prostate	convoluted, long, isodiametric	widened proximally, pyriform, short, arranged in a loop
penis	long, thin	short, conical
**Egg mass**		
shape	bean-shaped, transversally assymetrical	rounded, transversally symmetrical
dorsal keel	broad (0.8–1 mm)	narrow (0.4 mm)
**Substrate**	gorgonians (*Primnoisis antarctica*)	hydrozoans (*Oswaldella* sp., *Antarctoscyphus* sp.)

The egg clutches of *D*. *carinova* n. sp. are more elongated, slightly asymmetrical in transverse section, and possess a higher dorsal keel (1 mm) than those of *D*. *antarctica*. However, there are no histological differences in the mucus layers of the egg masses of the two species. Egg masses of both species were found in different cnidarian substrates, being perhaps different prey items. While *D*. *antarctica* was found on hydrozoans of the genera *Antarctoscyphus* and *Oswaldella*, *D*. *carinova* n. sp. was found on the gorgonian *Primnoisis antarctica*.

### Phylogenetic analysis

**Fig 7:**
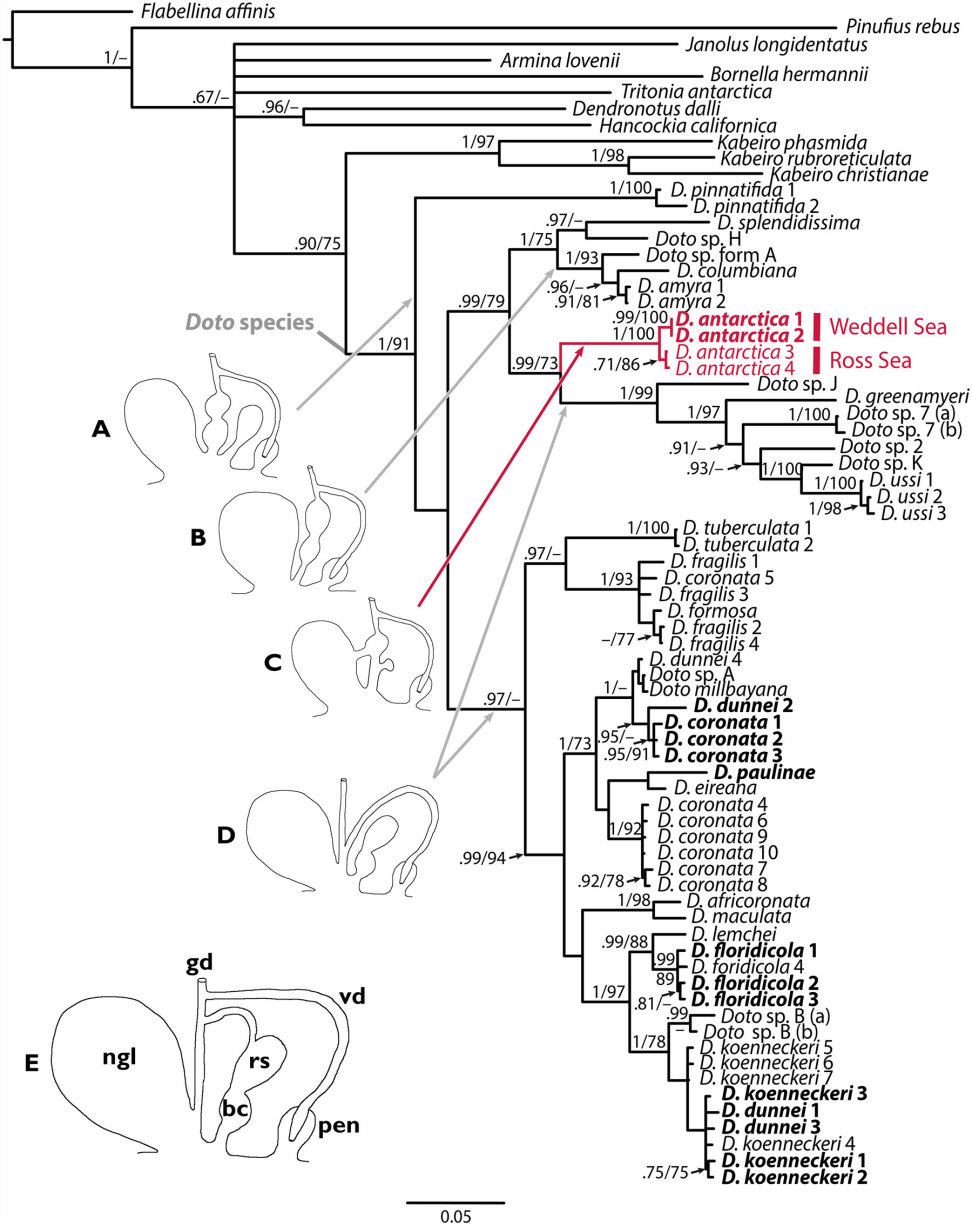
Phylogenetic tree of *Doto* species based on the combined COI, 16S, and H3 genes using Bayesian inference (BI) and maximum-likelihood (ML). Numbers on nodes indicate posterior probability values (BI) and bootstrap support values (ML). Specimens sequenced are in bold; *Doto antarctica* specimens are coloured in red. Schematic drawings of the reproductive system of *Doto* species are depicted (A–D), as well as the unsequenced *D*. *uva* Marcus, 1955 (E). *bc* bursa copulatrix; *gd* gonoduct; *nigl* nidamental glands; *pen* penis; *rs* receptaculum seminis; *vd* vas deferens.

The total data set contained 62 species of *Doto* and 11 outgroup species. The aligned genes comprised 2,283 characters. The maximum likelihood (ML) and Bayesian (BI) trees are similar (Fig 7). The family Dotidae resulted monophyletic. *Kabeiro* species are the sister group of all *Doto* species. Within *Doto*, *D*. *pinnatifida* was sister group to all other species. The two newly sequenced specimens of *D*. *antarctica* from the Weddell Sea clustered together and with the specimens from the Ross Sea. *Doto antarctica* is sister group of Philippine and Papua New Guinea specimens (*D*. *ussi*, *D*. *greenmayeri*, and other unidentified species). Thus *D*. *antarctica* is more closely related to Pacific *Doto* species than to Atlantic (northern and southern hemispheres) species.

Regarding the additional Mediterranean species sequenced herein, all of them clustered with species from the Northern Sea and Eastern North Atlantic. *Doto floridicola* specimens clustered with the Mediterranean specimen accessed in GenBank [44]. The specimens of *D*. *koenneckeri* grouped with the previous Mediterranean and Welsh specimens. However, two supposed *D*. *dunnei* specimens (#1 and #3) used in this study clustered also within the *D*. *koenneckeri* individuals. An additional *D*. *dunnei* (2) from the same locality was not found to be related to the previous specimen sequenced from Wales, but instead clustered with the three Mediterranean specimens of *D*. *coronata* sequenced here. However, these latter specimens did not cluster to any *D*. *coronata* specimens sequenced from the Netherlands, North Sea, USA, nor Wales. Although *D*. *coronata* has been also recorded in the Mediterranean, it is possible that this species is an undescribed species of *Doto*, since it presents morphological characters that differ from those of the original description (author’s unpub. data). Finally, *D*. *paulinae* was found to be closely related to *D*. *eireana*, although with low support.

## Discussion

In this study, we added molecular evidence for the circumpolar distribution of *D*. *antarctica*, as well as a detailed anatomical and histological description of the species and its egg masses. A thorough anatomical description is highly important since the species of *Doto* have often been misidentified due to the lack of clear external diagnostic characters. External descriptions available until now for *D*. *antarctica* clearly coincide with the description presented herein [17,19]. However, Eliot noted a ridge in front of the rhinophoral sheaths, which might be a fixation artefact [19]. Also, the number of rows of radular teeth in our dissected specimen was smaller than in previous observations [17,19], although this may be an age-dependent character. The shape of the rachidian tooth is consistent with the descriptions within the genus [45]. Since radulae are variable within species and among teeth of the same radula (usually bilaterally asymmetrical), this character is not considered relevant for distinguishing *Doto* species [9,12].

Histological sections of *D*. *antarctica* showed the typical notal epithelium of cladobranchs, composed by multivacuolised cells and mucus glandular cells [46,47]. The former cells protect the slug against cnidocysts [48], the latter are rather typical for Dendronotida [47,49]. Subepithelial clusters of single gland cells staining light blue (white in live specimens) were found in the most exposed parts of the animal, *i*.*e*., cerata and rhinophores. We propose a defensive function of these glands due to the strategic location in exposed parts, and because they everted their content when molested. These single glandular cells are commonly suggested to be defensive glandular cells [47,50], where the animal stores defensive compounds obtained from prey or *de novo* biosynthesised by the slug, such as terpenoids [51].

*Doto carinova* Moles, Avila & Wägele n. sp. was found in sympatry with *D*. *antarctica* in the Weddell Sea and firstly described herein. Although sequencing of the new species from chemically fixed material was impossible, morphological characters help to differentiate both species. Externally, *D*. *carinova* n. sp. exhibits a paler colouration, a higher number and more elongated cerata, and calyciform rhinophoral sheaths (see remarks above). Remarkably, the egg masses of both species described herein are unique in shape among *Doto* species. Usually they are ribbon like structures deposited on the substrate in a zig-zag folded way [15,16,52–54], whereas in the Antarctic species they have a bean-like shape, possessing a keel, and being attached to the substrate by a twisted stalk. However, the egg mass of *D*. *carinova* n. sp. is more pronouncedly reniform and has a wider keel. Furthermore, both species were found on different cnidarian species, albeit being collected from the same locality in the Weddell Sea. Different substrates might represent different food sources for the species, a fact that should be considered as an additional character for distinguishing among species [16]. Descriptions presented herein will be useful to identify the undetermined species of *Doto* collected in different Antarctic regions [18,22,24], and probably to expand the distribution of *D*. *carinova* n. sp.

Regarding the internal anatomy, the shape and arrangement of the salivary glands, ampulla, prostate, and penis are clearly diagnostic among both Antarctic species (see remarks above). A proximal blind diverticulum of the ampulla was only present in *D*. *antarctica*. The prostate is shorter and wider in *D*. *antarctica*, while it is longer in *D*. *carinova* n. sp. Both species present sphincters to separate ampulla, oviduct, and the allosperm vesicles (receptaculum seminis and bursa copulatrix), similarly to *D*. *uva* [54] and many other *Doto* species. The general outline of the reproductive system in *D*. *carinova* n. sp. is similar to *D*. *antarctica*. The diaulic reproductive system of both Antarctic species presents an oviduct leading into the receptaculum seminis (and bursa copulatrix annexed), which connects into a separate folded area of the nidamental glands (Fig 7C). Although the connection of the oviduct with the nidamental glands lies more internally in the Antarctic species, this arrangement of ducts is very similar to the more closely related *D*. *amyra* and *D*. *columbiana* (Fig 7B) [12]. A similar arrangement can be found in several other *Doto* species not included in our phylogenetic analyses (*D*. *bella*, *D*. *caramella*, *D*. *chica*, *D*. *divae*, *D*. *doerga*, *D*. *ganda*, *D*. *japonica*, *D*. *kya*, *D*. *varians*, and *D*. *wara* [12,50,55,56]). Another feature in which the Antarctic species differ from the latter species is the distinct bursa copulatrix attached to the vaginal duct.

*Doto pinnatifida* was recovered basal in our phylogenetic tree, displaying a similar arrangement to the Antarctic species, but with a bursa inserting close to the vaginal opening (Fig 7A) [57]. In addition, a fourth, quite spread, reproductive system is depicted; this displays an oviduct not connected proximally to the receptaculum (Fig 7D). This fourth system is found in a lineage close to *D*. *antarctica* and composed by *D*. *greenmayeri* and *D*. *ussi* [13]. Likewise, a second linage represented by *D*. *africoronata*, *D*. *coronata*, *D*. *floridicola*, *D*. *formosa*, *D*. *fragilis*, and *D*. *paulinae* also presented this system [13,56–62]. Additionally, according to Fischer *et al*. [54] a triaulic reproductive system is present in *D*. *uva* (Fig 7E), albeit Marcus [55] considered it similar to that of *D*. *pinnatifida*. Summarizing, our phylogenetic analyses show a trend towards the reduction of bursa copulatrix and the separation of the vaginal duct from the oviduct (Fig 7). This implies that the “flow through system” of the oviduct into vaginal duct is changing into a separate outleading duct for eggs and a shift of the fertilization chamber towards the distal part of the female genital system.

Histological analysis revealed gigantic gland cells around the salivary glands of *D*. *bella*, *D*. *japonica*, and *D*. *uva* [50,54]. They have been traditionally considered accessory glandular cells to the salivary glands. In our histological and tomographic analyses these giant cells resemble neuronal cells, similar to ganglionic cortical neurones, but larger in size. Moreover, these are not exclusively located close to the salivary glands but are in close contact to the ganglionic complex (see Fig 2C and 2D). Therefore, we consider them to be giant neurones as recorded in anaspideans and pulmonates [63], as well as in cladobranch and doridacean nudibranchs [64]. They are located in the ganglionic mass (*i*.*e*., metacerebral cells), related to external sensory input from the head and considered homologous within these groups [63]. These giant neurones have been found to be polyploid by increasing the DNA content step-wise as the animal grows [65]. This has been suggested to be related to a major hormone secretory function, responsible for behavioural responses such as crawling [64]. However, in *D*. *antarctica* and *D*. *carinova* n. sp., although being connected to the cerebropleural ganglia, they form a circle extending posteriorly (Fig 5). Since our GCs, like other described giant neurones, possess a huge (active) nucleus, we speculate that neurosecretory hormones might be secreted into the hemolymphatic sinus located within this circle. Our study seems to be the first description of a complex and asymmetrical neuronal/secretory arrangement in heterobranchs. Furthermore, it is possible that the number of GCs could be used as a diagnostic character for discriminating among *Doto* species. For instance, Antarctic species of *Doto* have larger (170 μm) and more abundant (27–40) GCs than the South American *D*. *uva* (150 μm, N = 12) [54].

The phylogenetic analyses recovered trees with similar topologies than others recently published [13,66]. However, contrary to Pola and Gosliner [6], *Pinufius* is not part of the Dotidae clade. *Doto antarctica* specimens from the Weddell Sea were closely related to those from the Ross Sea, as suggested morphologically herein. Contrary to the morphological similitudes mentioned by Eliot [17] when describing *D*. *antarctica*, and comparing to *D*. *fragilis* (Forbes, 1838), phylogenetic analyses revealed that these species are not closely related. Based on external appearance, Odhner [19] stated later that *D*. *antarctica* was more closely related to *D*. *formosa* Verrill, 1875. However, in our analyses, *D*. *fragilis* and *D*. *formosa* were closely related to each other, sharing a similar reproductive system, but were dissimilar to *D*. *antarctica*. Once more, descriptions merely based on external characters seem insufficient to establish phylogenetic relationships, or even to identify and describe *Doto* species. A closer phylogenetic relationship was found among *D*. *antarctica* and the Indo-Pacific species, and altogether with the rest of Southern species of *Doto*. This relationship could be related to the Antarctic origin of the group, and the older origin of the Indo-Pacific species respect to the Atlantic ones. However, the majority of the sequenced species so far are from the northern hemisphere. Thus, there is a need for sequencing more species from the Austral oceans to assess possible phylogeographic relationships among *Doto* species. Further sampling efforts should be conducted to collect and sequence *D*. *carinova* n. sp. and other undetermined species around Antarctica to increase the knowledge of Dotidae, with only two species found in the Southern Ocean to date.

## Concluding Remarks

New Dotidae species have been usually described based only on external morphological and radular characters. Nonetheless, internal organ organisation and egg mass structure is desirable for describing *Doto* species. Micro-CT and histology has demonstrated to be very useful techniques to reconstruct the internal anatomy of these two *Doto* species. Two new occurrences of *D*. *antarctica* were recorded in Bouvet Island and the eastern Weddell Sea. These specimens are morphologically and genetically characterised herein and appeared related to *D*. *antarctica* from the Ross Sea, which strongly suggests a circumpolar distribution. We also described *D*. *carinova* n. sp. occurring in sympatry with *D*. *antarctica* in the Weddell Sea. Although some distinguishing characters can be size-related, the lower number of tubercles on the cerata, the different form of the rhinophoral sheath, the shape and arrangement of the salivary glands, ampulla, and prostate in the large specimen of *D*. *carinova* n. sp., as well as differences in the egg masses and cnidarian substrate indicate separate evolutionary lineages.

A phylogenetic hypothesis including various species of *Doto* from various regions showed a trend towards the reduction of bursa copulatrix and distal connection of oviduct to the nidamental glands with separate pathways for eggs and allosperm. Furthermore, we identified and described the nervous system of *Doto* species that contains accessory giant cells that might represent neurones with neuronal/secretory function. Future studies may unravel the properties and function of these peculiar giant cells. Moreover, further studies should revisit and check the identification of *Doto* species collected in former Antarctic cruises, because the present study provides new characters that may allow distinguishing among species.

## Supporting Information

S1 3D PDF Reconstructed anatomy of the anterior region of *Doto antarctica*. It can be opened in Adobe Acrobat Reader and activated by clicking on it. Each system and organ can be selected independently.
(PDF)

S2 3D PDF Reconstructed anatomy of the anterior region of *Doto carinova* Moles, Avila & Wägele n. sp. It can be opened in Adobe Acrobat Reader and activated by clicking on it. Each system and organ can be selected independently.
(PDF)

S1 Table Accession numbers of the species included in the phylogenetic analysis. Species sequenced in this study are in bold.
(DOCX)
