# Supplementary figures and images for: The End of the Cold Loneliness: 3D Comparison between Doto antarctica and a New Sympatric Species of Doto (Heterobranchia: Nudibranchia)
Source: PLoS One. 2016 Jul 13;11(7):e0157941. doi: 10.1371/journal.pone.0157941 (PMC4943632; doi:10.1371/journal.pone.0157941)

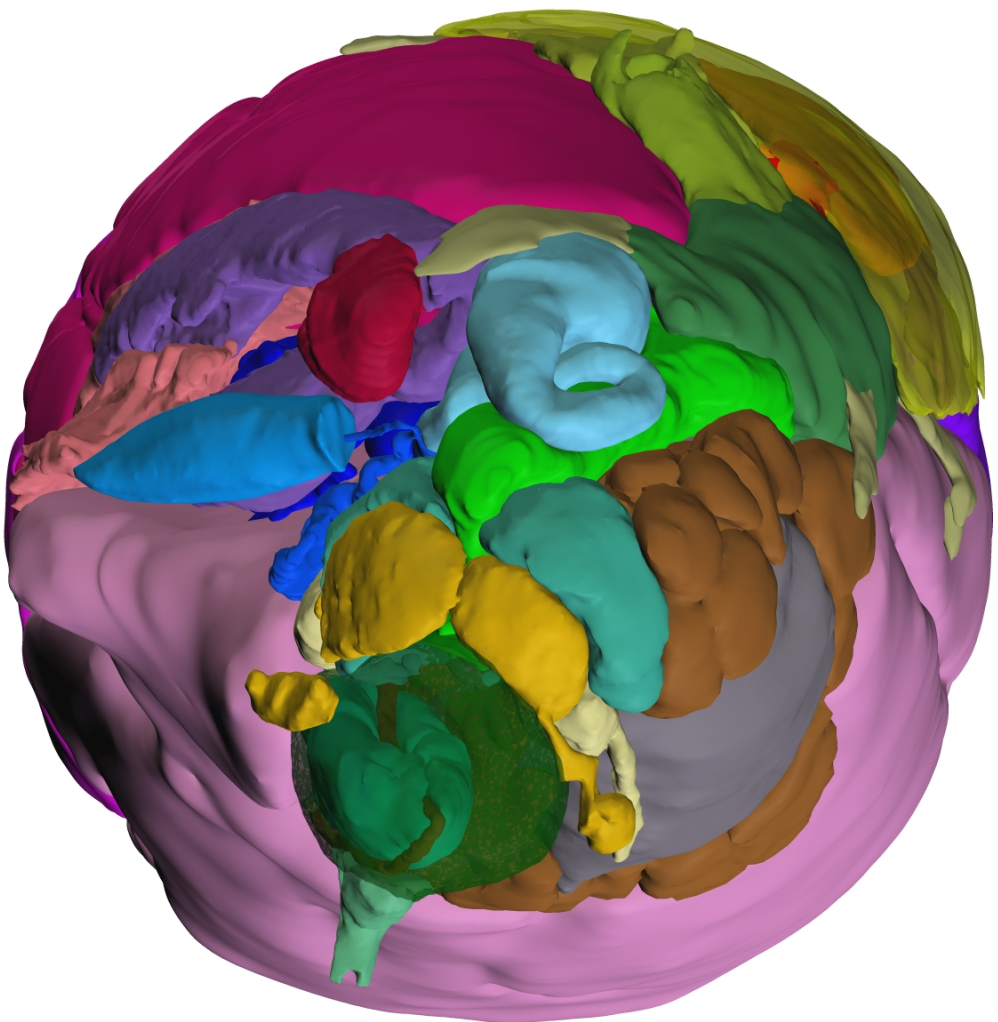

Supplement: S1 3D PDF — It can be opened in Adobe Acrobat Reader and activated by clicking on it. Each system and organ can be selected independently. (PDF) [file pone.0157941.s001.pdf]

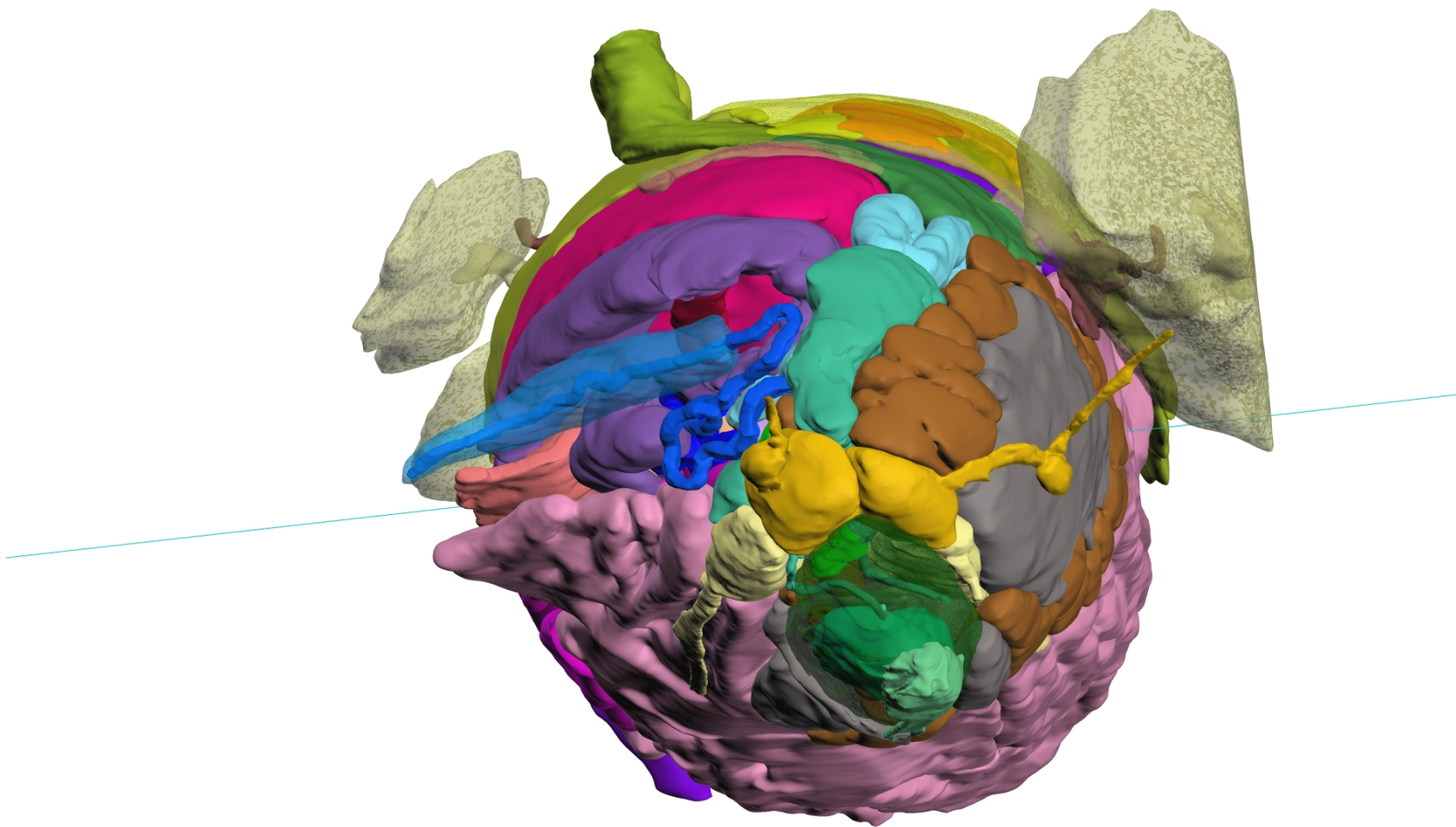

Supplement: S2 3D PDF — It can be opened in Adobe Acrobat Reader and activated by clicking on it. Each system and organ can be selected independently. (PDF) [file pone.0157941.s002.pdf]
