# Supplementary material for: The End of the Cold Loneliness: 3D Comparison between Doto antarctica and a New Sympatric Species of Doto (Heterobranchia: Nudibranchia)
Source: PLoS One. 2016 Jul 13;11(7):e0157941. doi: 10.1371/journal.pone.0157941 (PMC4943632; doi:10.1371/journal.pone.0157941)
Supplement: S1 Table — Species sequenced in this study are in bold. (DOCX) [file pone.0157941.s003.docx]

**The end of the cold loneliness: 3D comparison between *Doto antarctica* and a new sympatric species of *Doto* (Heterobranchia: Nudibranchia)**

Juan Moles,^1,*^ Heike Wägele,^2^ Manuel Ballesteros,^1^ Álvaro Pujals,^1^ Gabriele Uhl,^3^ Conxita Avila^1^

^1^Department of Evolutionary Biology, Ecology and Environmental Sciences and Biodiversity Research Institute (IrBIO), University of Barcelona, Av. Diagonal 645, 08028 Barcelona, Catalonia, Spain

^2^Zoologisches Forschungsmuseum Alexander Koenig, Adenauerallee 160, 53113 Bonn, Germany

^3^General and Systematic Zoology, Zoological Institute and Museum, University of Greifswald, Anklamer Str. 20, Greifswald 17489, Germany

***Corresponding author**: moles.sanchez@gmail.com

Telephone: + 34 93 402 0161, Fax: +34 93 403 5740

**Supplementary Table S1.** Species included in the phylogenetic analysis. Species sequenced in this study are in bold.

| ***Species*** | **Locality** | **Voucher** | **CO1** | **16S** | **H3** | **Reference** |
| --- | --- | --- | --- | --- | --- | --- |
| *Armina lovenii* | Kattegat, North Sea |  | AF249781 | AF249243 | - | Wollscheid-Lengeling *et al.*, 2001 |
| *Bornella hemannii* | Malaysia: Tokong Kamundi South | CASIZ175743 | HM162705 | HM162625 | HM162531 | Pola & Gosliner, 2010 |
| *Dendronotus dalli* | USA, North Atlantic |  | AF249800 | AF249252 | - | Wollscheid-Lengeling *et al*., 2001 |
| *Doto africoronata* | South Africa | CASIZ176278 | HM162734 | HM162657 | HM162566 | Pola & Gosliner, 2010 |
| *Doto amyra* 1 | California, USA | CASIZ179473b | KJ486702 | KJ486767 | KJ486670 | Shipman & Gosliner, 2015 |
| *Doto amyra* 2 | California, USA | CASIZ181213 | KJ486703 | KJ486768 | KJ486674 | Shipman & Gosliner, 2015 |
| ***Doto antarctica* 1** | **N Kapp Norvegia, E Weddell Sea, Antarctica** | **ANTXV/3**  **48/033-91** | **KX274295** | **KX274324** | **KX274308** | **This study** |
| ***Doto antarctica* 2** | **N Kapp Norvegia, E Weddell Sea, Antarctica** | **ANTXV/3**  **48/033-92** | **KX274294** | **KX274325** | **KX274310** | **This study** |
| *Doto antarctica* 3 | Ross Sea, Antarctica |  | GQ292025 | - | - | Shields *et al*., 2009 |
| *Doto antarctica* 4 | Ross Sea, Antarctica | CASIZ190213 | KJ486705 | KJ486765 | KJ486686 | Shipman & Gosliner, 2015 |
| *Doto columbiana* | Washington, USA |  | GQ292026 | - | - | Shields *et al*., 2009 |
| ***Doto coronata* 1** | **Blanes, Spain, Mediterranean Sea** | **CBL1** | **KX274285** | **KX274321** | **KX274305** | **This study** |
| ***Doto coronata* 2** | **Blanes, Spain, Mediterranean Sea** | **CBL5** | **KX274287** | **KX274323** | **KX274306** | **This study** |
| ***Doto coronata* 3** | **Palamós, Spain, Mediterranean Sea** | **CFO2** | **KX274286** | **KX274322** | **KX274307** | **This study** |
| *Doto coronata* 4 | Kattegat, North Sea |  | AF249794 | - | - | Wollscheid-Lengeling *et al*., 2001 |
| *Doto coronata* 5 | North Sea |  | KR084788 | - | - | Barco *et al*., 2016 |
| *Doto coronata* 6 | Eastern Scheldt, Netherlands | CASIZ190710a | KJ486720 | KJ486763 | KJ486655 | Shipman & Gosliner, 2015 |
| *Doto coronata* 7 | Pembrokeshire, Wales | Mn33146 | KJ486722 | KJ486764 | KJ486652 | Shipman & Gosliner, 2015 |
| *Doto coronata* 8 | Tuskar Rock, Skomer, Wales | Mn33135 | KJ486723 | KJ486762 | KJ486653 | Shipman & Gosliner, 2015 |
| *Doto coronata* 9 | Eastern Scheldt, Netherlands | CASIZ190710b | KJ486721 | KJ486761 | KJ486656 | Shipman & Gosliner, 2015 |
| *Doto coronata* 10 | Maine, USA | CASIZ183936 | KJ486719 | KJ486760 | KJ486654 | Shipman & Gosliner, 2015 |
| ***Doto dunnei* 1** | **Cap de Creus, Spain, Mediterranean Sea** | **DPG1** | **KX274292** | **KX274318** | **KX274300** | **This study** |
| ***Doto dunnei* 2** | **Cap de Creus, Spain, Mediterranean Sea** | **DPG2** | **KX274293** | **KX274319** | **KX274301** | **This study** |
| ***Doto dunnei* 3** | **Cap de Creus, Spain, Mediterranean Sea** | **DME3** | **KX274291** | **KX274320** | **KX274299** | **This study** |
| *Doto dunnei* 4 | Pembrokeshire, Wales | Mn33147 | KJ486725 | - | KJ486659 | Shipman & Gosliner, 2015 |
| *Doto eireana* | Spain, NE Atlantic | CASIZ190544 | KJ486657 | AF249248 | - | Wollscheid-Lengeling *et al*., 2001; Shipman & Gosliner, 2015 |
| ***Doto floridicola*** **1** | **Cap de Creus, Spain, Mediterranean Sea** | **FTN4** | **KX274290** | **KX274313** | **KX274303** | **This study** |
| ***Doto floridicola* 2** | **Cap de Creus, Spain, Mediterranean Sea** | **FTN5** | **KX274288** | **KX274312** | **KX274302** | **This study** |
| ***Doto floridicola* 3** | **Cap de Creus, Spain, Mediterranean Sea** | **FTN6** | **KX274289** | **KX274314** | **KX274304** | **This study** |
| *Doto floridicola* 4 | Spain, Mediterranean Sea |  | AF249820 | - | - | Wollscheid-Lengeling *et al*., 2001 |
| *Doto formosa* | Maine, USA | CASIZ183923 | - | - | KJ486667 | Shipman & Gosliner, 2015 |
| *Doto fragilis* 1 | Sweeden |  | - | AJ223392 | - | Thollesson, 1999 |
| *Doto fragilis* 2 | North Sea |  | KR084559 | - | - | Barco *et al*., 2016 |
| *Doto fragilis* 3 | Ferrol, Spain: Atlantic Coast |  | - | KJ486754 | - | Shipman & Gosliner, 2015 |
| *Doto fragilis* 4 | Pembrokeshire, Wales | Mn33151 | KJ486735 | KJ486755 | KJ486668 | Shipman & Gosliner, 2015 |
| *Doto greenamyeri* | Papua New Guinea | CASIZ185101 | KJ486715 | KJ486769 | KJ486683 | Shipman & Gosliner, 2015 |
| ***Doto koenneckeri* 1** | **Palamós, Spain, Mediterranean Sea** | **KFO5** | **KX274283** | **KX274316** | **KX274297** | **This study** |
| ***Doto koenneckeri* 2** | **Palamós, Spain, Mediterranean Sea** | **KFO6** | **KX274284** | **KX274315** | **KX274296** | **This study** |
| ***Doto koenneckeri* 3** | **Palamós, Spain, Mediterranean Sea** | **KFO7** | **KX274282** | **KX274317** | **KX274298** | **This study** |
| *Doto koenneckeri* 4 | Spain, NE Atlantic |  | AF249797 | AF249249 | - | Wollscheid-Lengeling *et al*., 2001 |
| *Doto koenneckeri* 5 | Thorn Rock, Skomer, Wales | Mn33141 | KJ486732 | KJ486752 | KJ486665 | Shipman & Gosliner, 2015 |
| *Doto koenneckeri* 6 | Thorn Rock, Skomer, Wales | Mn33140 | KJ486730 | KJ486751 | KJ486666 | Shipman & Gosliner, 2015 |
| *Doto koenneckeri* 7 | Mediterranean Sea | CASIZ176815 | KJ486729 | KJ486750 | KJ486664 | Shipman & Gosliner, 2015 |
| *Doto lemchei* | Thorn Rock, Skomer, Wales | Mn33144 | KJ486727 | KJ486749 | - | Shipman & Gosliner, 2015 |
| *Doto maculata* | Pembrokeshire, Wales | Mn33143 | - | KJ486757 | KJ486661 | Shipman & Gosliner, 2015 |
| *Doto millbayana* | Tuskar Rock, Skomer, Wales | Mn33145 | KJ486726 | KJ486759 | KJ486660 | Shipman & Gosliner, 2015 |
| ***Doto paulinae*** | **Mataró, Spain, Mediterranean Sea** | **J33-1** | **KX274281** | **KX274311** | **KX274309** | **This study** |
| *Doto pinnatifida* 1 | Spain, NE Atlantic |  | AF249797 | AF249250 | - | Wollscheid-Lengeling *et al*., 2001 |
| *Doto pinnatifida* 2 | Tuskar Rock, Skomer, Wales | Mn33137 | KJ486736 | KJ486748 | KJ486689 | Shipman & Gosliner, 2015 |
| *Doto* sp. 2 | Philippines | CASIZ177543 | HM162737 | HM162660 | HM162569 | Pola & Gosliner, 2010 |
| *Doto* sp. 7 (a) | Philippines | CASIZ177542 | HM162738 | HM162661 | HM162570 | Pola & Gosliner, 2010 |
| *Doto* sp. 7 (b) | Philippines | CASIZ181291 | KJ486711 | KJ486771 | KJ486685 | Shipman & Gosliner, 2015 |
| *Doto* sp. A | Thorn Rock, Skomer, Wales | Mn33136 | KJ486724 | KJ486758 | KJ486658 | Shipman & Gosliner, 2015 |
| *Doto* sp. B (a) | Açores Islands, Portugal | CASIZ178247 | HM162735 | HM162658 | HM162567 | Pola & Gosliner, 2010 |
| *Doto* sp. B (b) | Açores: Sao Miguel Island | CASIZ178248 | KP940456 | KP940451 | KP940461 | Shipman & Gosliner, 2015 |
| *Doto* sp. form A | California, USA | CASIZ182040 | KJ486704 | KJ486766 | KJ486673 | Shipman & Gosliner, 2015 |
| *Doto* sp. H | Mexico | LACM174964 | HM162740 | HM162663 | HM162572 | Pola & Gosliner, 2010 |
| *Doto* sp. J | Sardinia, Italy | CASIZ175711 | HM162742 | HM162665 | HM162574 | Pola & Gosliner, 2010 |
| *Doto* sp. K | Philippines | CASIZ177460 | HM162575 | HM162666 | HM162575 | Pola & Gosliner, 2010 |
| *Doto splendidissima* | South Africa | CASIZ176123 | HM162742 | HM162664 | HM162573 | Pola & Gosliner, 2010 |
| *Doto tuberculata* 1 | Pembrokeshire, Wales | Mn33142 | KJ486734 | KJ486756 | KJ509924 | Shipman & Gosliner, 2015 |
| *Doto tuberculata* 2 | Spain: Atlantic Coast | CASIZ190542 | KJ486733 | - | KJ486669 | Shipman & Gosliner, 2015 |
| *Doto ussi* 1 | Philippines | CASIZ177438 | HM162736 | HM162659 | HM162568 | Pola & Gosliner, 2010 |
| *Doto ussi* 2 | Philippines | CASIZ182893 | KJ486706 | KJ486780 | KJ486675 | Shipman & Gosliner, 2015 |
| *Doto ussi* 3 | Philippines | CASIZ177514 | KP940457 | KP940452 | KP940462 | Pola & Gosliner, 2015 |
| *Flabellina affinis* | Menorca, Spain, Mediterranean Sea | MNCN15.05/53696 | HQ616753 | HQ616716 | HQ616782 | Carmona *et al*. 2011 |
| *Hancockia californica* | Costa Rica | CASIZ175722 | HM162702 | HM162621 | HM162257 | Pola & Gosliner, 2010 |
| *Janolus longidentatus* | South Africa: A-Frame: Western False Bay, Cape Prov. Philippines: | CASIZ176320 | HM162749 | HM162673 | HM162582 | Pola & Gosliner, 2010 |
| *Kabeiro christianae* | Philippines | CASIZ185993 | - | KJ486782 | KJ486691 | Shipman & Gosliner, 2015 |
| *Kabeiro phasmida* | Philippines | CASIZ177545 | HM162739 | HM162662 | HM162571 | Pola & Gosliner, 2010 |
| *Kabeiro rubroreticulata* | Philippines | CASIZ177726 | KJ486739 | KJ486791 | KJ486697 | Shipman & Gosliner, 2015 |
| *Pinufius rebus* | Philippines | CASIZ177763 | HM162744 | HM162667 | HM162576 | Pola & Gosliner, 2010 |
| *Tritonia antarctica* | Bouvet Island, Sub-Antarctica | CASIZ171177 | HM162718 | HM162643 | HM162550 | Pola & Gosliner, 2010 |

**References**

Barco A, Raupach MJ, Laakmann S,  Neumann H, Knebelsberger T. Identification of North Sea molluscs with DNA barcoding. Mol Ecol Res. 2016;16: 288-297.

Carmona L, Gosliner TM, Pola M, Cervera JL. A molecular approach to the phylogenetic status of the aeolid genus *Babakina* Roller, 1973 (Nudibranchia). J Molluscan Stud. 2011;77: 417–422.

Pola M, Gosliner TM. The first molecular phylogeny of cladobranchian opisthobranchs (Mollusca, Gastropoda, Nudibranchia). Mol Phylogenet Evol. 2010;56: 931–941.

Pola M, Gosliner TM. A new large and colourful species of the genus *Doto* (Nudibranchia: Dotidae) from South Africa. J Nat Hist. 2015;49: 2465–2481.

Shields CC. Nudibranchs in the Ross Sea, Antarctica: Lineage diversity and divergence estimated using methods of molecular phylogenetics and sequence divergence. Unpublished thesis, Clemson University, Clemson, South Carolina. 2009.

Shipman C, Gosliner T Molecular and morphological systematics of *Doto* Oken, 1851 (Gastropoda: Heterobranchia), with descriptions of five new species and a new genus. Zootaxa. 2015;3973: 57–101.

Thollesson M. Phylogenetic analysis of dorid nudibranchs (Gastropoda : Doridacea) using the mitochondrial 16S rRNA gene. J Molluscan Stud. 1999;65: 335–353.

Wollscheid-Lengeling E, Boore J, Brown W, Wägele H. The phylogeny of Nudibranchia (Opisthobranchia, Gastropoda, Mollusca) reconstructed by three molecular markers. Org Divers Evol. 2001;1: 241–256.
